# Supplementary material for: Vaccination-related attitudes and behavior across birth cohorts: Evidence from Germany
Source: PLoS One. 2022 Feb 14;17(2):e0263871. doi: 10.1371/journal.pone.0263871 (PMC8843242; doi:10.1371/journal.pone.0263871)
Supplement: S1 Table — Non-imputed and (last column) multiply imputed KIGGS data. (PDF) [file pone.0263871.s001.pdf]

S1 Table. Descriptive Statistics. Non-imputed and (last column) multiply imputed KIGGS data.

Variable	Original data					Imputed data
	Obs.	Mean/%	Min.	Max.	% missing	Mean/%
1st Measles at 24 months	14.007		0	1	0.0	
no		26.2				26.2
yes		73.8				73.8
Deliberate reasons	13.784		0	1	1.6	
no deliberate reasons		92.6				92.7
1+ deliberate reason(s)		7.4				7.3
Convenience reasons	13.784		0	1	1.6	
no convenience reasons		98.6				98.6
1+ convenience reason(s)		1.4				1.4
Cohort	14.007		1	4	0.0	
1987-1990		21.8				21.8
1991-1994		26.6				26.6
1995-1998		27.7				27.7
1998-2002		23.8				23.8
Sex of child	14.007		0	1	0.0	
male		51.0				51.0
female		49.0				49.0
Migration	13.920		0	1	0.6	
native		80.9				80.8
migrant		19.1				19.2
Size of town	14.007		0	1	0.0	
<100T		50.8				50.8
100T+		49.2				49.2
Region	14.007		0	1	0.0	
West Germany (& Berlin)		68.7				68.7
East Germany (w/o Berlin)		31.3				31.3
Education parents	13.882		1	3	0.9	
no/low/med. sec. degr.		4.8				4.9
high sec. degr./voc. tr.		49.3				49.4
higher tertiary degree		45.9				45.7
Siblings	13.677		0	1	2.4	
no older (half) siblings		46.4				46.2
1+ older (half) siblings		53.6				53.8
Age mother	13.786		0	1	1.6	
older		16.5				16.4
mother <36 yrs. when c. 2 yrs.		83.5				83.6
Age first in nonpar. supervision	13.775		0	98	1.7	
at age 0 [0]		3.2				3.1
at age 1 [1]		14.9				15.0
at age 2 [2]		13.7				13.7
at age 3 [3]		34.0				33.9
at age 4 to 6 [4]		15.7				15.7
never [98]		18.5				18.5
