# Supplementary material for: Vaccination-related attitudes and behavior across birth cohorts: Evidence from Germany
Source: PLoS One. 2022 Feb 14;17(2):e0263871. doi: 10.1371/journal.pone.0263871 (PMC8843242; doi:10.1371/journal.pone.0263871)
Supplement: S2 Table — Odds-Ratios from logistic regressions using weighted and multiply imputed data; McFadden’s R2 derived using Rubin’s combination rules and ignoring the clustered data structure; Significance: + p<0.10, * p<0.05, ** p<0.01. (PDF) [file pone.0263871.s002.pdf]

S2 Table. Regression estimates of deliberate and convenience reasons to not vaccinate depicted in Fig 3. Odds-Ratios from logistic regressions using weighted and multiply imputed data; (1) McFadden's R^2 derived using Rubin's combination rules and ignoring the clustered data structure; Significance: + p<0.10, * p<0.05, ** p<0.01.

	Deliberate reason(s)	Convenience reason(s)
Cohort (ref.: 1987-1990)		
1991-1994	0.87	0.83
1995-1998	0.66**	0.63+
1998-2002	0.57**	0.39**
Migrant	0.43**	0.59+
Large town 100T+	1.19+	1.15
East Germany (w/o Berlin)	0.24**	0.72
Education parents (ref.: no/low/med. secondary degree)		
high secondary degree/vocational training	2.02*	0.37**
higher tertiary degree	4.39**	0.32**
1+ older (half) siblings	1.04	1.81**
Age mother <36 years when child 24 m.	0.68**	0.73
McFadden's R^2 (1)	0.073	0.028
$N_{imputed}$	14,007	14,007
