# Supplementary material for: Vaccination-related attitudes and behavior across birth cohorts: Evidence from Germany
Source: PLoS One. 2022 Feb 14;17(2):e0263871. doi: 10.1371/journal.pone.0263871 (PMC8843242; doi:10.1371/journal.pone.0263871)
Supplement: S3 Table — Odds-Ratios from logistic regressions using weighted and multiply imputed data; McFadden’s R2 derived using Rubin’s combination rules and ignoring the clustered data structure; Nimputed: due to the imputation of some missing values on the reason variables the estimation sample differ slightly between the 25 sets of imputed data; Significance: + p<0.10, * p<0.05, ** p<0.01. (PDF) [file pone.0263871.s003.pdf]

S3 Table. Regression estimates of having received first measles vaccination at age of 24 month for three groups depicted in Fig 5. Odds-Ratios from logistic regressions using weighted and multiply imputed data; (1) McFadden's R^2 derived using Rubin's combination rules and ignoring the clustered data structure; (2) $N_{imputed}$: due to the imputation of some missing values on the reason variables the estimation sample differ slightly between the 25 sets of imputed data; Significance: + p<0.10, * p<0.05, ** p<0.01.

	With deliberate reason(s)	With convenience reason(s)	No reasons
Cohort (ref.: 1987-1990)			
1991-1994	0.77	1.15	1.28**
1995-1998	0.51**	0.29*	1.74**
1998-2002	0.47**	0.60	3.21**
Female	1.20	1.16	1.13*
Migrant	0.90	1.25	0.71**
Large town 100T+	0.98	1.35	1.06
East Germany (w/o Berlin)	1.17	4.18*	2.14**
Education parents (ref.: no/low/med. sec. degr.)			
high secondary degree/vocational training	1.24	67.71**	1.53**
higher tertiary degree	0.71	47.81**	1.62**
1+ older (half) siblings	0.70*	0.37+	0.57**
Age mother <36 years when child 24 m.	1.28	1.15	1.02
Non-parent. sup. (ref.: never)			
at age 0 [0]	0.90	0.30	0.89
at age 1 [1]	1.59	0.31+	1.04
at age 2 [2]	0.70	0.41	1.16
at age 3 [3]	0.98	0.90	1.11
at age 4 to 6 [4]	1.36	1.47	1.11
McFadden's R^2 (1)	0.053	0.139	0.050
$N_{imputed}$ (2)	1,023 - 1,032	188 - 195	12,868 - 12,877
