# Supplementary material for: Vaccination-related attitudes and behavior across birth cohorts: Evidence from Germany
Source: PLoS One. 2022 Feb 14;17(2):e0263871. doi: 10.1371/journal.pone.0263871 (PMC8843242; doi:10.1371/journal.pone.0263871)
Supplement: S4 Table — Odds-Ratios from logistic regressions using weighted and multiply imputed data; (1) McFadden’s R2 derived using Rubin’s combination rules and ignoring the clustered data structure; Significance: + p<0.10, * p<0.05, ** p<0.01. (PDF) [file pone.0263871.s004.pdf]

S4 Table. Regression estimates of vaccination behavior depicted in Fig 6. Odds-Ratios from logistic regressions using weighted and multiply imputed data; (1) McFadden's R^2 derived using Rubin's combination rules and ignoring the clustered data structure; Significance: + p<0.10, * p<0.05, ** p<0.01.

	Odds Ratio
Cohort (ref.: 1987-1990)	
1991-1994	1.29**
1995-1998	1.76**
1998-2002	3.23**
Female	1.14**
Migrant	0.73**
Large town 100T+	1.05
East Germany (w/o Berlin)	2.08**
Education parents (ref.: no/low/med. Sec. degr.)	
high secondary degree/vocational training	1.54**
higher tertiary degree	1.64**
1+ older (half) siblings	0.58**
Age mother <36 years when child 24 m.	1.04
Non-parent. sup. (ref.: never)	
at age 0 [0]	0.89
at age 1 [1]	1.08
at age 2 [2]	1.10
at age 3 [3]	1.10
at age 4 to 6 [4]	1.14+
Interactions with deliberate & convenience reasons	
Deliberate reason(s)	0.83
Convenience reason(s)	0.02**
Deliberate reas. # 1991-1994	0.56**
Deliberate reas. # 1995-1998	0.29**
Deliberate reas. # 1998-2006	0.14**
Convenience reas. # 1991-1994	1.21
Convenience reas. # 1995-1998	0.24*
Convenience reas. # 1998-2006	0.28+
Deliberate reas. # high sec. degr./voc. tr.	0.75
Deliberate reas. # higher tertiary degree	0.37
Convenience reas. # high sec. degr./voc. tr.	38.37**
Convenience reas. # higher tertiary degree	51.61**
McFadden's R^2 (1)	0.100
$N_{imputed}$	14,007
