# Supplementary figures and images for: Vaccination-related attitudes and behavior across birth cohorts: Evidence from Germany
Source: PLoS One. 2022 Feb 14;17(2):e0263871. doi: 10.1371/journal.pone.0263871 (PMC8843242; doi:10.1371/journal.pone.0263871)

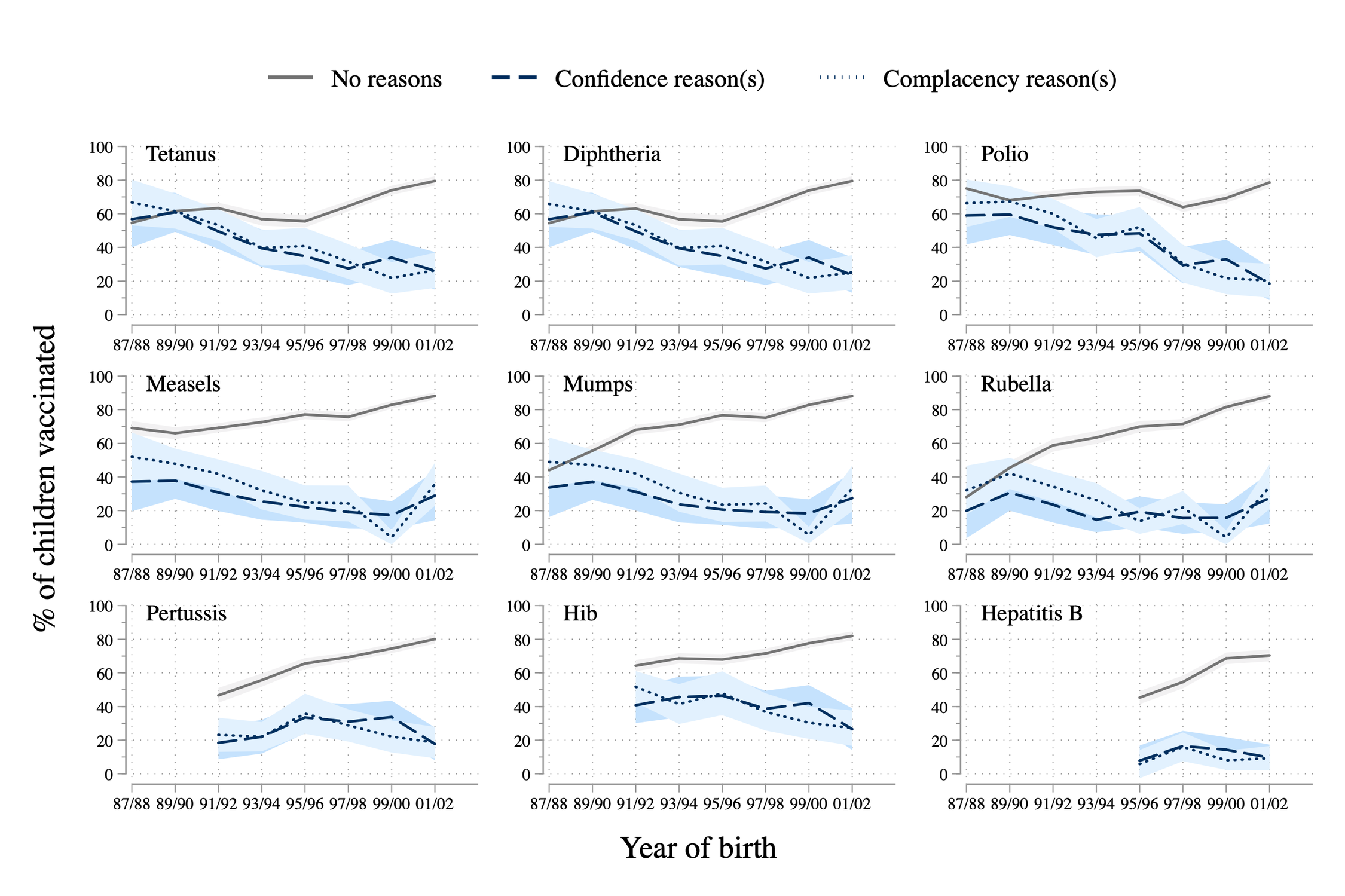

Supplement: S1 Fig — (TIF) [file pone.0263871.s005.tif]

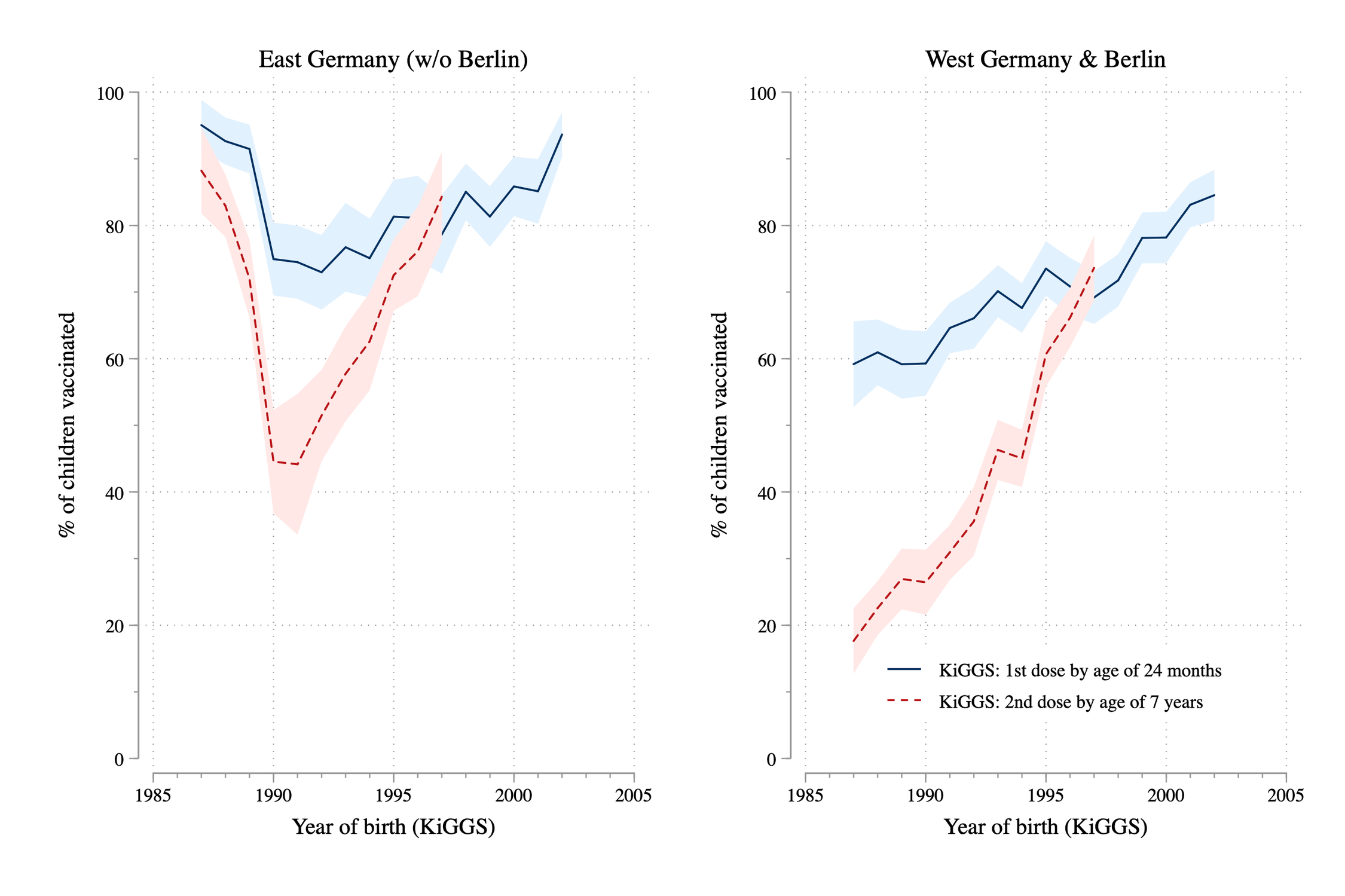

Supplement: S2 Fig — KiGGS data weighted, 14,007 observations for first measles dose by age 24 months and 9,715 observations for second dose by age 7 years, point estimates and 95 percent confidence intervals. (TIF) [file pone.0263871.s006.tif]

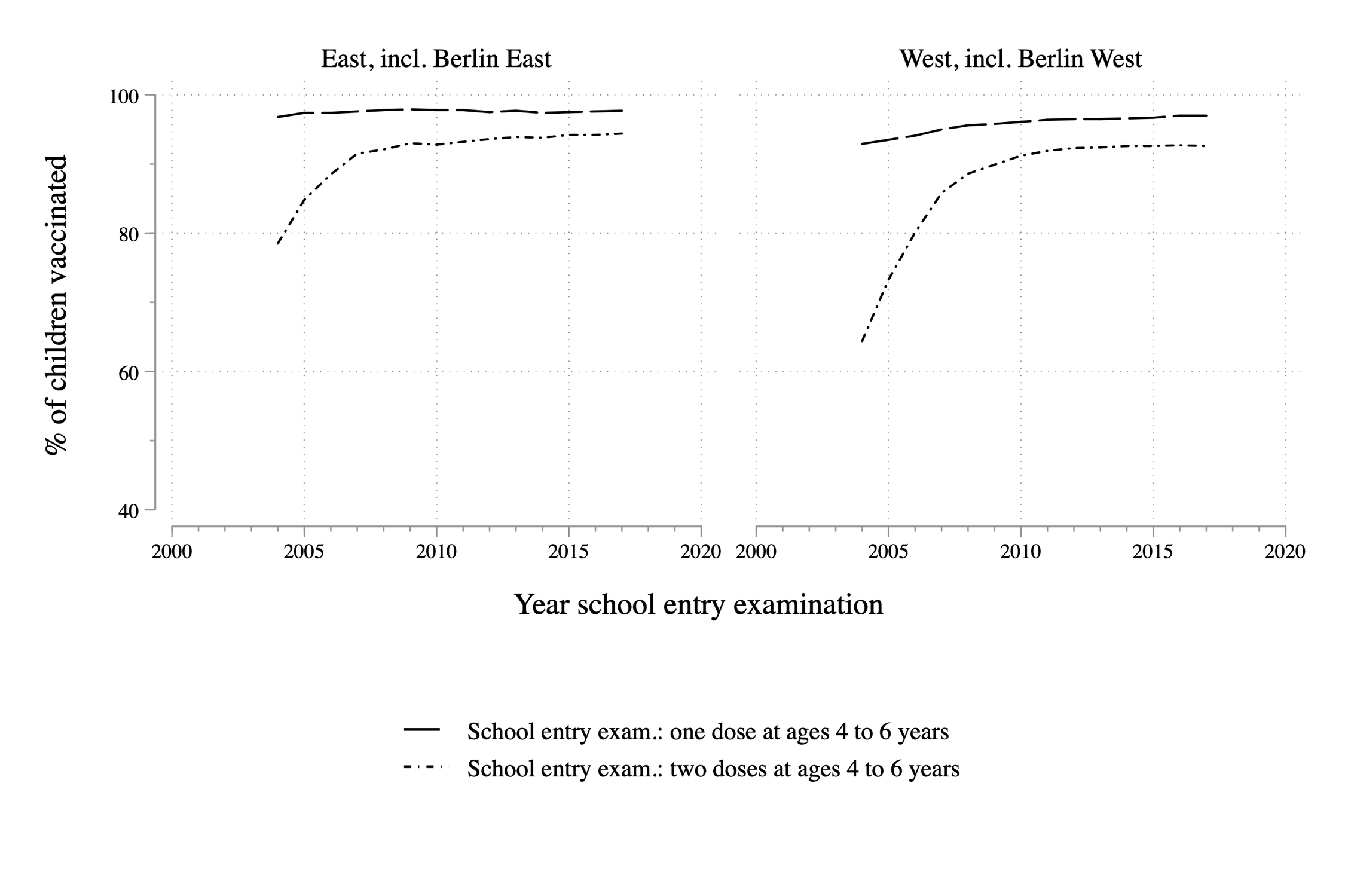

Supplement: S3 Fig — (TIF) [file pone.0263871.s007.tif]

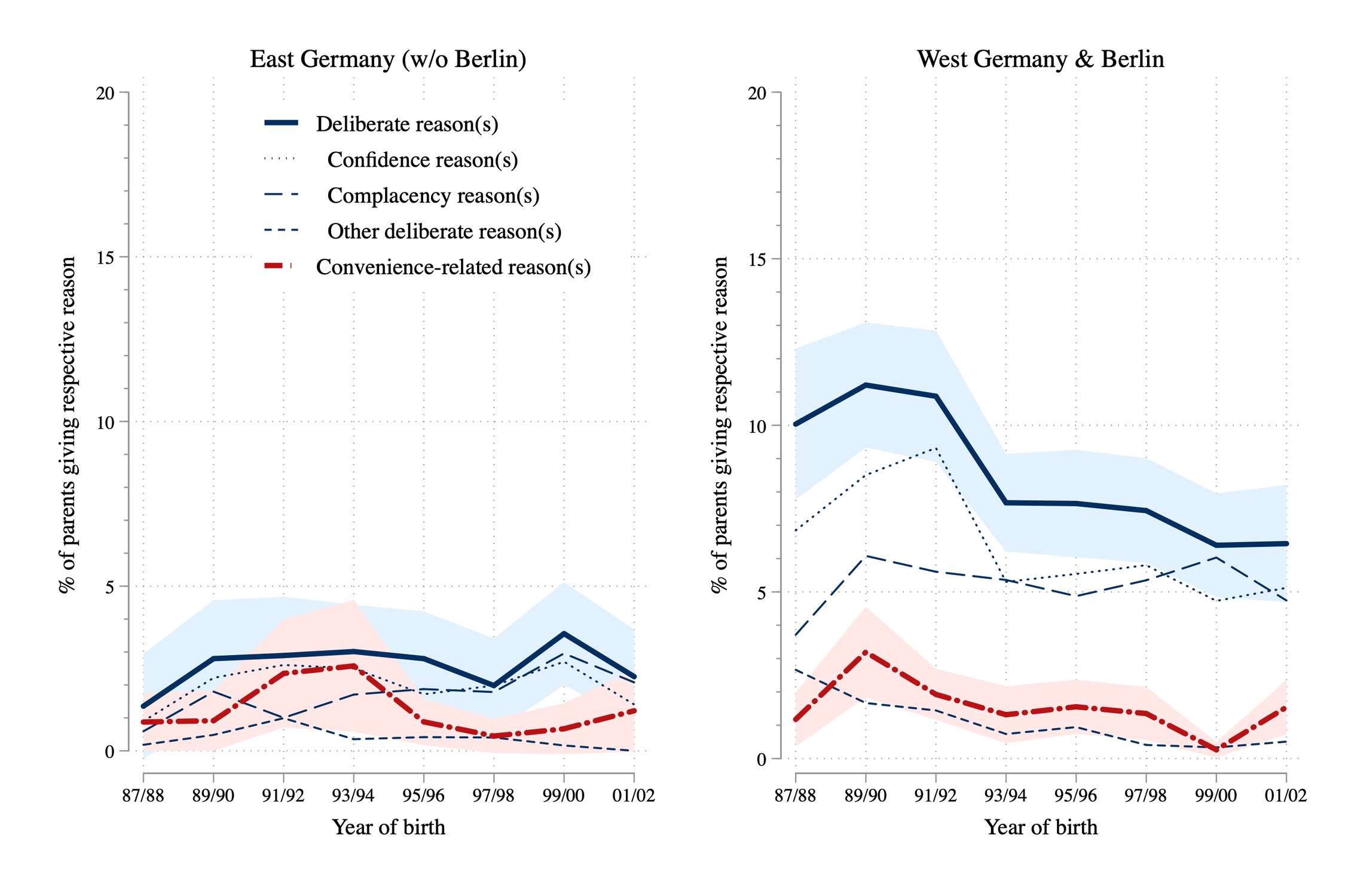

Supplement: S4 Fig — KiGGS data weighted, depending on reason between 13,517 and 13,784 observations, point estimates and 95 percent confidence intervals. (TIF) [file pone.0263871.s008.tif]

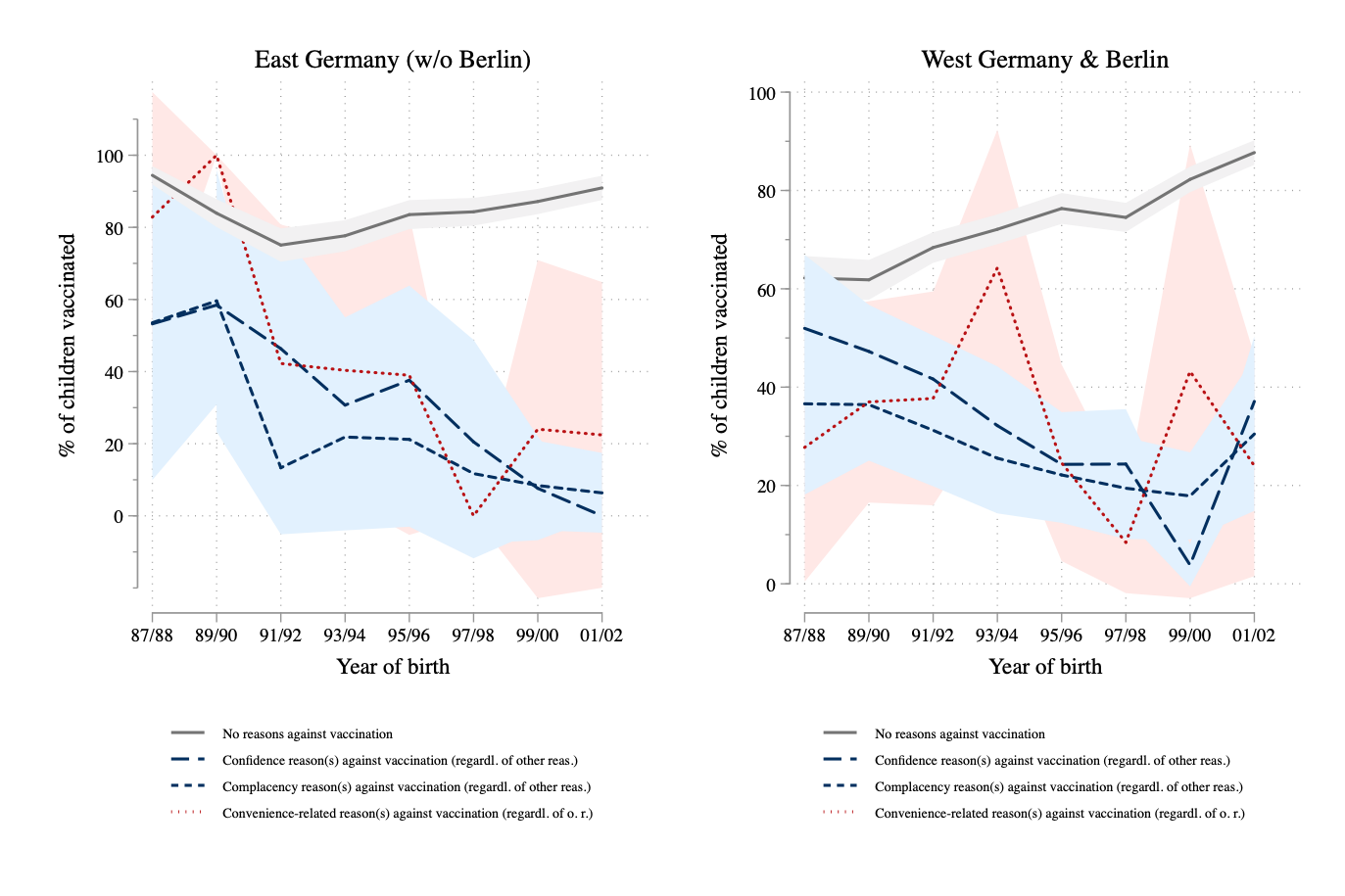

Supplement: S5 Fig — KiGGS data weighted, point estimates and 95 percent confidence intervals. (TIF) [file pone.0263871.s009.tif]
