# Supplementary material for: Vaccination-related attitudes and behavior across birth cohorts: Evidence from Germany
Source: PLoS One. 2022 Feb 14;17(2):e0263871. doi: 10.1371/journal.pone.0263871 (PMC8843242; doi:10.1371/journal.pone.0263871)
Supplement: Attachment — Submitted filename: Response to Reviewers.docx [file pone.0263871.s010.docx]

Vaccination-related attitudes and behavior across birth cohorts. Evidence from Germany

Dear colleagues,

thank you very much for the detailed and extensive feedback on our paper. We very much appreciate the helpful comments and suggestions. By now, we have thoroughly revised our paper. We document all changes below and also elaborate on the few instances in which we were not able or willing to follow your recommendations.

Best regards,

Claudia Diehl, and Christian Hunkler

| Reviewer | Response |
| --- | --- |
| **Editor** |  |
| Writing should be proofread by a **native English speaker** due to the linguistic and grammatical errors. | The revised manuscript was edited by an official language editor (native-English speaker). |
| **Reviewer 1** |  |
| (…). Even if the data they collected **shows that independent of the internet attitudes and behavior change over time, this** **does not allow to judge the influence the internet has on vaccination behavior and attitudes**. It might have a strong influence – and other factors might also have a strong influence. The reviewer suggests to discuss this more explicitly and to shorten the discussions about potential influence of the internet in the manuscript. | Thank you for pointing out that our argument about the role of the internet reads too much as a general comment about the irrelevance of the internet in explaining vaccine-hesitancy. That was certainly not our intention. In the revised version we shortened the text accordingly and changed the headline that was, in fact, suggesting that generally the internet does not matter for vaccine hesitancy. We also clarified that the trend *we describe* is unrelated to the internet because almost no one used it during that time. We thus drop all general claims about the role of the internet. |
| The reviewer further suggests that the authors discuss more in dept, which other factors could play a role in changes in vaccination behavior and attitudes. E.g. one hypothesis they have is that **health education** has improved. Do they have any evidence to prove that? It would be interesting to read how the time period 1990-2000 has been in Germany with regard to health education. | We incorporate the (helpful) suggestion to elaborate on the possible role of health education/ literacy in the revised “Discussion and limitation” section. |
| The authors should include a paragraph under **methods** where they describe the **regression analysis in detail**. This should include that they used logistic regression, that the dependent variables were dichotomous (e.g. 0= not vaccinated; 1= vaccinated), that all analysis was done with Stata?, how they built the regression models, interactions, whether they looked for **multicollinearity**. They should further explain how they sorted the participants in different **groups** according to the reasons they gave for non-vaccination, e.g. an individual who gives both confidence and complacency reason, it is included in both subgroups then? | We restructured the “Data and Methods” sections to make it more accessible to readers.  We added more information on the details of the regression analysis and on the robustness checks. We describe how we defined the subgroups of respondents with different reasons for non-vaccination in greater detail in a separate section (“Groups”). We checked all reported models for multicollinearity, the VIF index is 1.15 at most (obviously the variables included in interaction terms show higher VIFs, but that does not affect the interaction coefficients or their p-values). The groups indeed overlap as supposed by Reviewer 1, but only a very small fraction of parents (N= 82, i.e., 0.6% of the analysis sample) gave deliberate as well as convenience reasons. They are indeed included in both subgroups. We also provide more details on the interaction terms in the analysis section and placed the reference to the Appendix tables more prominently in the text. |
| The authors should discuss that those with “no reasons” probably do have reasons for non-vaccination, but that these reasons were not assessed. Moreover, it is difficult to generalize on vaccine attitudes for the whole sample from this data, e.g. a claim like the data “contains valid information on changes across cohorts in parents’ confidence in vaccination, their vaccination complacency” seems difficult to make, as they do not have data on confidence for the whole sample (p. 26 line 21). E.g. Betsch et al. developed a measure that assess confidence among all participants, thus e.g. a mean confidence for a given birth cohort could be reported. | The items used in KiGGS are not perfect for our purposes. Unfortunately, the KiGGS study was conducted years before the development of the 3C model, and hence does not include validated measures for confidence in vaccinations. We now discuss this in greater detail in the Data & Methods section and mention it in the new paragraph on limitations.  However, the reasons for non-vaccination were indeed assessed for the whole sample. *All* respondents – including parents whose children received all vaccinations - were asked “Did you have reasons for not having immunizations given to your child?”. We double checked this with the KiGGS study group. We highlighted this prominently in the “Data and Methods” section in the revision.  As we understand, reviewer 1 is also concerned about those parents whose children received all vaccinations even though the parents had reservations about vaccinations. For those parents the question may indeed have sounded somewhat awkward. However, the share of observed children with all recommended vaccinations applied at age 24 months is only 36% and drops to only 14.5% when vaccinations recommended at age 7 years are considered. The majority (70%) of interviews took place when the child was age 7 or older. So, the share of observations affected by the question wording is rather low.  When revising our manuscript, we also looked closely into the relationship between reporting reasons for not having immunizations given to the child and the actual vaccination behavior. In doing so, we used the information on all nine recommended vaccinations. We found a substantial number of children who had received all recommended vaccinations in time but whose parents still reported reasons against vaccinations - and vice versa.  In sum, we believe this concern is justified, but in the end affects only a minority of the KiGGS sample. |
| The reviewer is not convinced that attitudes stay as stable over time – could the authors provide more evidence here to back their methodology? Moreover, even though vaccination decisions are more frequent in early childhood, vaccination is not a topic for small children only. E.g. considering the TDaP booster, the FSME vaccine, the MenB vaccine for travelers. | We see this point but we are afraid that there is not much we can do about it – except for being very transparent about it. In the revised text, we argue more clearly that it is unlikely that the vaccinations applied later in life (like the ones mentioned by reviewer 1) shape vaccination attitudes. We add that people get these vaccinations completely voluntarily and that there is no public debate or pressure surrounding them. While the literature suggests that anti-vaccination attitudes are difficult to change, there is no data on when in the life course these attitudes evolve. We just have to stick here with our argument that it is very likely that parents make up their mind about vaccinations when they have small children and doctors tell them that time has come to vaccinate them. |
| “whether or not parents with deliberate reasons against vaccination have become more likely across birth cohorts to have unvaccinated children”. How did the authors develop this hypothesis? How did the authors develop this hypothesis? Why did they come to focus on this research question in the first place? Was this an exploratory analysis or an hypothesis with which they approached the research project? Maybe the authors could make this clearer in the manuscript. | In the revised introduction we clarify that our analyses on the link between attitudes and behavior are exploratory in nature and follow previous analyses by Poethko-Mueller (2019). We state the aims of our paper more clearly (see next comment) and streamlined them with the empirical section of the paper.  *“Against this backdrop, our paper seeks to make a modest yet substantive contribution to the existing knowledge about trends in vaccination-related attitudes and behavior—and the link therein. Using data from the German KiGGS survey [6], we describe first, whether and in which direction vaccination behavior has changed across cohorts and second, whether a low confidence in vaccination and/or vaccination complacency is particularly prevalent among parents whose children belong to more recent birth cohorts. Based on this, we explore, third, changes in vaccination behavior separately for parents who did or who did not report any reasons against vaccinations in the KiGGS survey. This is important because an overall trend of increasing vaccination rates may conceal the possibility that the subgroup of parents who are hesitant about vaccinations may have become more likely to have unvaccinated children. While previous analyses used KiGGS data to describe the vaccination rates of children whose parents have reasons against vaccination [7], those studies did not examine the changes across birth cohorts.”* |
| The reviewer suggests to shorten the introduction and place the hypothesis/ research questions in one place for better readability of the manuscript. To the reviewer it was difficult to grasp the research questions as they seemed to occur twice in the introduction and seemed to vary. | In the revised version of the manuscript, we substantially shortened the introduction and framed the research questions more clearly at the end of the introduction. We clarify that the current debate about the COVID-19 vaccination raises the question of whether what we see is the culmination of a long-term trend towards more vaccine hesitancy. We then state that this question is impossible to answer due to a lack of trend data on vaccine hesitancy. And then we explain our aims (see previous comment). |
| The reviewer suggests to include a limitations section under discussion. …This section should, among others, include that – as the authors note – (i) no longitudinal data was available, the method they chose is an approximation of what happens over time, (ii) comment on the way they assessed vaccine hesitancy (e.g. no validated items, items that may not capture the dimensions confidence, complacency, convenience really well, because they were used before the research field developed a clear concept of vaccine hesitancy), (iii) that this is data which captures a time period in the 1990s, thus does not allow to extrapolate a trend in the development of vaccine hesitancy in recent years, (iv) attitudes were assessed at one point in time (2003-2006) but parents should comment on their vaccination behavior a long time ago, not at the point in time where they took the decision, (v) the by far largest group was a group of individuals who did not give reasons for non-vaccination, thus for the largest groups it remains unknown why they chose not to vaccinate. | We added a separate paragraph on limitations in the section that is now called “Discussion and Limitations” and systematically discusses all of these limitations. In this section, we incorporate the problem that for those parents who did not give any reasons against vaccinations it remains unclear why they did not vaccinate their children. |
| The authors mention institutional trust or mistrust and its relationship with vaccine hesitancy in the introduction. However, it is unclear how this relates to their findings, as they do not assess institutional trust in their study. | As social scientists we do not want to give up the link to political polarization but we shortened this part in both the intro and the discussion/conclusion sections of the paper. In doing so, we are very explicit about the limitation that trust in the government was not measured in the KiGGS study. |
| The authors link their results to COVID-19 and the current interest in vaccine hesitancy in the verge of the pandemic. The review suggests to shorten this part, as it does not lead the reader well to measles vaccination behavior and attitudes 20-30 years ago. | We shortened this part and briefly discuss the link between our findings on vaccination hesitancy against measles, the current situation and political polarization. |
| The authors should mention the surveys on vaccination attitudes and behaviors conducted by the Federal Centre for Health Education. This is cross-sectional data on vaccination behavior and attitudes during the last 5-10 years. https://www.bzga.de/forschung/studien/abgeschlossene-studien/studien-ab-1997/impfen-und-hygiene/ | We have incorporated some findings from these studies in the (relabeled) section “The 3C model and Existing Findings on Vaccine Hesitancy” |
| p. 4 line 7. The authors should replace this reference with a more recent reference, covering vaccination coverage at school entry 2008-2018. Rieck et al. 2020 https://edoc.rki.de/bitstream/handle/176904/6902.4/32-33_2020_DOI_Impfquoten_Version%203.pdf?sequence=9&isAllowed=y | We added the reference. |
| The authors might want to change the threshold for herd immunity (60-70 percent for Covid-19). To the reviewer’s knowledge no such threshold is currently known and if there was one it is estimated to be higher than 60-70 percent. | Oh well. Yes! Following the suggestion above, we shortened the discussion of our findings in the light of the ongoing pandemic. Given the fact that new mutations keep showing up, we decided to refrain from presenting any precise herd immunity threshold… |
| The authors might want to change the terminology from vaccination hesitancy to vaccine hesitancy.  p. 9 line 11. The authors might want to consider a more recent publication on physician recommendation behavior: Neufeind et al. 2020. https://www.sciencedirect.com/science/article/pii/S0264410X20305466 | We changed the terminology accordingly and also want to the thank the reviewer for the link to this interesting article that we now quote. |
| The survey was conducted between May 2003 and May 2006. The reviewer would like to know the authors thoughts about whether this is methodologically a problem, as attitudes might have changed between 2003 and 2006? It the authors think so they might want to consider to add this under limitations. | We do not think that the long field period constitutes a problem, given that in this period no “critical events” happened. To rule out any concern, we repeated the analysis by splitting up the sample by survey year and re-estimated the multivariate logistic regressions shown in Figures 4 and 6 in the paper (see end of this letter). Only few coefficients change direction, overall the pattern remains pretty stable, though confidence bands widen. |
| p.12 line 15. The authors write that they “shifted the upper horizontal axis five years to the left to roughly “match” with the rates calculated from the KiGGS data that are displayed by birth cohort”. The reviewer has difficulty understanding what the authors did here and why. Maybe they could add further explanation for better understanding? How do the authors account for the difference in the age groups between KiGGS (24 months and 7 years) and school entry exam (4-6 years)? | We added a more precise and extensive explanation on school entry exam data:   “*Whereas the KiGGS data record the vaccination status of children by their year of birth, the vaccination rates from the school entry examination refer to about 5 year old children. A child born in 2000 will thus appear in the school entry examination data approximately in 2005. To account for this, we shifted the upper horizontal axis five years to the left to match with the rates calculated from the KiGGS data that are displayed by year of birth*.“ |
| The authors generally in the figures report the sample size of e.g. those with no reasons, they do not give the sample size for each birth cohort. The is probably to keep it simple, however thus the reader has a skewed understanding of the confidence intervals. Maybe the authors could think of a way to give the reader an understanding of the sample size in the respective birth cohorts? | We complemented the figures with information on the range of observations each data point is based on. We do this either in the legend or caption of each figure, to not “overload” them with information. |
| The group with the category **low**est **education** seems to be very **small**. Is this groups underrepresented in the sample? If so, how did the authors address this problem? Was this a problem in their regression models? | This category may seem small at first sight, but note that we used the highest degree of schooling achieved by either mother *or* father. This implies that only children are included in the low education category whose mother *and* father have low levels of education.  Still, surveys participation is typically lower in the population with low levels of education. This is why all analyses are weighted. This accounts for sampling design and also for non-response. Specifically, education is among the marginal distributions used to estimate the response probability. |
| Could the authors explain why **time intervals chosen varied**? The first time intervals seem to be four years long, the last one eight years? Does it make a difference in the results when those intervals are changed? | The first time intervals are exactly 4 years long (1987-1990, 1991-1994, 1995-1998). The last one is actually also four years long (1998-2002), but in some figures and tables we accidently had labelled it with 1998-2006 – thank you for catching this.  This typo is a relic of an earlier version of the paper, in which we had also analyzed children born between 2003 and 2006. However, these are the years the KiGGS data was collected, and vaccination status is only available for children who were older than 24 months. Therefore, we dropped these birth cohorts from the analyses (see the paragraph “KiGGS analysis sample” in “Data and Methods”). |
| The manuscript might benefit from a **language check** and could be written in a more concise way. | The revised manuscript was edited by an official language editor (native-English speaker). |
| **Reviewer 2:** |  |
| From what I understand, parents were asked the reasons for not vaccinating their children. The same question was asked of parents of young and older children. No comment is made about the potential recall bias associated with parents of older children being asked to comment on reasons for not vaccinating when those decisions were many years ago. How has this been accounted for in the analysis? | We discuss this possibility more extensively in the revised version and mention recall bias explicitly. |
| The purpose of using the entry exam data is not clear and this data is not fully described or presented. | We fully describe the school entry examination data and its purpose in the revised “Data and Methods” section. In short, while not suitable for multivariate analyses, these data allow to describe trends in vaccinations rates to a much more recent time compared to the KiGGS data. Comparing KiGGS and entry exam data also reveals that many children are vaccinated against measles later than recommended. |
| The number of people providing different responses is not clear. Table 1 should have numbers also, not just %. | This is a very good point: we complemented Table 1 with the number of parents giving reasons. |
| Fig 5 is missing a full label for the y axis. % of what? | Even one of the authors had the same problem, when working on the revision, so we added full labels to all y axes shown in the paper and the supplementary information. |
| Sentence: "Living in a migrant family reduces the chances of being vaccinated for children whose parents do not report any reasons against vaccination". -- Compared to which group? The following sentence is also not clear. | We clarified this in the revised manuscript. |
| **Journal Requirements:** |  |
| Please include your full ethics statement in the ‘Methods’ section of your manuscript file. In your statement, please include the full name of the IRB or ethics committee who approved or waived your study, as well as whether or not you obtained informed written or verbal consent. If consent was waived for your study, please include this information in your statement as well. | We included a revised ethics statement, answering all points, in the subsection “Data” of the “Data and Methods” section:  “The KiGGS baseline study was approved by the ethics committee of Charité - Universitätsmedizin Berlin and by the Federal Commissioner for Data Protection. Participation was voluntary. Informed written consent was obtained from all parents and from adolescents aged 14 years and older.” |
| We note that you have stated that you will provide repository information for your data at acceptance. Should your manuscript be accepted for publication, we will hold it until you provide the relevant accession numbers or DOIs necessary to access your data. If you wish to make changes to your Data Availability statement, please describe these changes in your cover letter and we will update your Data Availability statement to reflect the information you provide. | We have to change our Data Availability Statement:  “The KiGGS data cannot be shared publicly because participants were assured that the data will only be used for scientific research and hence informed consent did only include restricted sharing of the data. The data ("KiGGS_0100_v2.dta") are available to researchers from the Research Data Center of the Robert Koch Institute (contact via [fdz@rki.de](mailto:fdz@rki.de), +49 (0)30 18754 3200, https://www.rki.de/rdc) for researchers who meet the criteria for access to confidential data.”  To facilitate replication, we made the Stata scripts to prepare the data and to replicate the analyses available via Open Science Framework:  <https://doi.org/10.17605/OSF.IO/36RB8> We added a short description to the Data and Methods section:  Data section: “The data used for this paper (“KiGGS_0100_v2”) are available to researchers via the Research Data Center of the Robert Koch Institute (contact via [fdz@rki.de](mailto:fdz@rki.de)).  Statistical Analysis section: “The scripts to prepare the data and replicate the analyses are available via an Open Science Framework repository (<https://doi.org/10.17605/OSF.IO/36RB8>).“ |

**Replication of Fig 4 and Fig 6 for single survey years**

**Average marginal effects from logistic regressions predicting deliberate and convenience-related reasons to not vaccinate by survey year.** Point estimates, 95 percent confidence intervals showing average marginal effects based on weighted data.

**Average marginal effects from three logistic regressions predicting vaccination of the child in different groups of parents by survey year.** Point estimates, 95 percent confidence intervals showing average marginal effects based on weighted data. For convenience reasons we cannot split the data by each survey year (too small cells) and therefore only compared 2003/4 and 2005/6.
